# A new LUCApedia database for data-driven research on early evolutionary history

**DOI:** 10.1093/bioadv/vbaf309

**Published:** 2025-12-04

**Authors:** Zahra Nikfarjam, Ishaan Thota, Alireza Nikfarjam, Freya Kailing, Aaron D Goldman

**Affiliations:** Department of Biology, Oberlin College, Oberlin, OH 44074, United States; Department of Biology, Oberlin College, Oberlin, OH 44074, United States; Department of Quantitative and Computational Biology, University of Southern California, Los Angeles, CA 90089, United States; Department of Computer Engineering, Islamic Azad University, Bushehr Branch, Bushehr, 1651153311, Iran; Department of Biology, Oberlin College, Oberlin, OH 44074, United States; Department of Computational Biology, Cornell University, Ithaca, NY 14853, United States; Department of Biology, Oberlin College, Oberlin, OH 44074, United States; Blue Marble Space Institute of Science, Seattle, WA 98104, United States

## Abstract

**Motivation:**

Many topics within the study of the origin and early evolution of life are amenable to computational research strategies. Over a decade ago, the original LUCApedia was developed in order to facilitate such research. Here we describe a massively overhauled LUCApedia database and web server.

**Results:**

The database is composed of 17 different datasets based on previous studies or published hypotheses about the last universal common ancestor and its evolutionary predecessors. Similar to the original LUCApedia database, these datasets are mapped onto a common framework so that they can be corroborated with one another and used to examine continuity across different stages of early evolution.

**Availability and implementation:**

The database can be searched, browsed, and downloaded from the LUCApedia web server, https://lucapedia.org/.

## 1 Introduction

All extant life on Earth is related to a Last Universal Common Ancestor (LUCA) (Woese 1998) that lived roughly 4 billion years ago (Betts *et al.* 2018, Moody *et al.* 2024). Genome and proteome reconstructions of the LUCA suggest that organisms at this time had already evolved many of the foundational characteristics typical of cellular life (Becerra *et al.* 2007, Goldman and Fournier 2024). Specifically, the LUCA was most likely a cellular organism (Gribaldo and Cammarano 1998, Harris and Goldman 2021, Kailing *et al.* 2024) that possessed a DNA genome (Goldman and Landweber 2012), a sophisticated translation system (Crick 1968, Fournier *et al.* 2011, Hsiao *et al.* 2009, Rivas and Fox 2024, Fer *et al.* 2025), and a complex metabolic network (Goldman *et al.* 2012, Ledford and Meredith 2024). Such a complex LUCA indicates that a significant amount of evolutionary change must have occurred between this stage and the origin of life.

While details about the origin of life continue to be debated, several distinct geochemical environments have been identified as possible settings for life’s emergence, such as hydrothermal vent systems (Baross and Hoffman 1985, Russell and Hall 1997, Martin *et al.* 2008), iron-sulfur mineral surfaces (Huber and Wächtershäuser 1997, Russell and Hall 1997, Wächtershäuser 1988), clays (Ferris *et al.* 1979, Ferris *et al.* 1996), and ices (Levy *et al.* 2000, Menor-Salvan *et al.* 2009), among others. In addition to a productive geochemical environment, the emergence of a system of replication with heredity would have been essential to any origin of life scenario.

One of the most influential ideas about an early system of heredity is the so-called “RNA World Hypothesis.” As it was originally conceived, the RNA World described a genetic system in which RNA genes encoded functional RNAs (Visser 1984, Gilbert 1986), thereby simplifying the current tripartite genetic system composed of DNA, RNA, and proteins. A pure RNA World genetic system has since fallen out of favor in large part due to the difficulty of synthesizing RNAs under geochemical conditions (De Duve 1988). However, recent studies have shown that the kinds of long RNAs required for an RNA World stage in early evolution can indeed be synthesized under prebiotically plausible conditions (Jerome *et al.* 2022). Some examples of more nuanced versions of an RNA World scenario include a co-evolution of RNAs with proteins (Bowman *et al.* 2015) or within protocellular compartments (Lai *et al.* 2021). While the specific character of an early RNA-based genetic system is unknown, evolutionary evidence strongly supports the notion that translation emerged from some sort of RNA-based genetic system (Rivas and Fox 2023), likely prior to the emergence of the DNA genome (Freeland *et al.* 1999, Goldman *et al.* 2010).

All of these scenarios can be investigated by using techniques from computational biology and bioinformatics. The LUCA has been studied for several decades by reconstructing its minimal genome or proteome (Becerra *et al.* 2007, Crapitto *et al.* 2022). A potential RNA World can be modeled by identifying extant protein enzymes that perform similar functions to artificial RNA catalysts (Cech 2002) or proteins that use extant coenzymes that may be relics of an RNA World (White 1976, Kirschning 2021). Similarly, earlier stages of prebiotic chemistry may be modeled based on the use of inorganic cofactors that may reflect the geochemical environment of life’s origin (Chu and Zhang 2020, Goldman and Kacar 2021). The LUCApedia database was designed over a decade ago to integrate these data onto a common framework, thereby facilitating computational research on ancient life. Here we describe a major update to the LUCApedia database, designed to represent a greater breadth of knowledge about the origin and early evolution of life.

## 2 The new LUCApedia database

The new LUCApedia database consists of 17 datasets that can be categorized as representing four distinct stages of early evolution: Prebiotic Chemistry, the RNA World, LUCA Protein Domains, and LUCA Protein Families. Each dataset within the four categories represents either the results of a single empirical study or a previously published hypothesis about the origin or early evolution of life.

Study-based datasets were created by mapping the results of the study (usually downloaded from supplemental data) onto protein accessions in the UniProt database (Huang *et al.* 2011, UniProt Consortium 2023). For example, a recent study on the proteome of the LUCA identified 399 likely protein families (Moody *et al.* 2024) represented by the KEGG Orthology (KO) database (Kanehisa *et al.* 2016). The LUCApedia dataset representing this study was created by mapping these KO accessions onto individual Uniprot accessions. Hypothesis-based datasets were created by identifying proteins in the UniProt database with the characteristics denoted by that hypothesis. For example, one popular hypothesis linking prebiotic chemistry to early proteins is that the iron-sulfur clusters found in proteins today are a relic of reactions occurring on iron-sulfur mineral surfaces that may have played an important role in the origin of life (Wächtershäuser 1988, Huber and Wächtershäuser 1997). The LUCApedia dataset representing this hypothesis was created by identifying and aggregating all proteins in the UniProt database (Boeckmann *et al.* 2003) known to use an iron-sulfur cluster cofactor. It is important to note, however, that while this and other potentially ancient cofactors are thought to have ancient roots in prebiotic chemistry or early evolutionary history, it is not the case that all proteins that use them are necessarily ancient in origin (Rivas *et al.* 2011).

**Figure 1.**
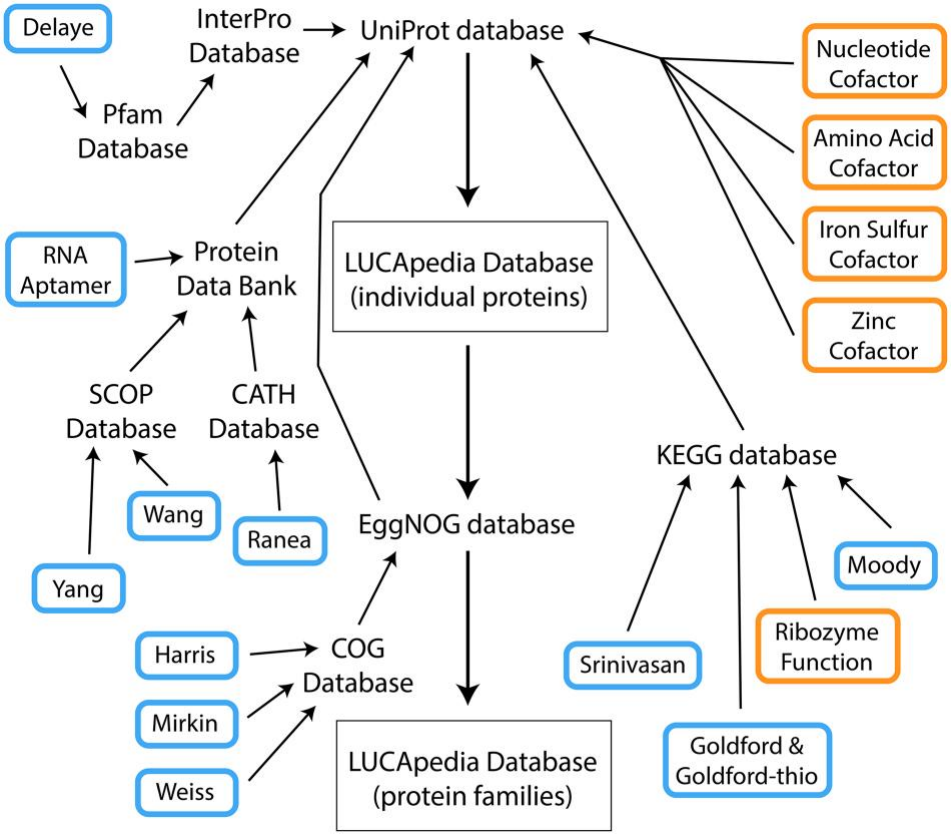
A flowchart of database mapping used to create the LUCApedia datasets. Study-based datasets, named after the first author of the study, involve mapping the predictions acquired from the study’s supplemental data onto UniProt database accessions (UniProt Consortium 2023). Hypothesis-based databases involve aggregating protein accessions in the UniProt database that have annotations associated with the hypothesis. A separate LUCApedia database centered on protein families was created by mapping UniProt accessions to clusters of homologous proteins defined by the EggNOG database (Huerta-Cepas *et al.* 2019).

In addition to mapping the predictions from these studies and hypotheses onto protein accessions in the UniProt database, they were also mapped onto protein families defined by the EggNOG database (Huerta-Cepas *et al.* 2019) (Fig. 1). In order to provide functional annotations for each LUCApedia entry, Gene Ontology (GO) terms were also linked to each UniProt accession and these GO terms were aggregated for all UniProt accessions in each EggNOG cluster. In all cases, this mapping between databases is never completely efficient due to the idiosyncrasies of each database and the ways in which protein functions or membership in a protein family is defined. For example, protein families as defined by the KEGG Ortholog (KO) database tend to be smaller than protein families defined by the EggNOG database, meaning that different KOs will often be assigned to the same COG.

Individual proteins may also be missing certain annotations that would place them within a LUCApedia dataset. For example, many individual proteins may perform a certain function or fold into a certain structure, but are not included in the relevant LUCApedia dataset because their functions are not annotated in UniProt or their structure has not been solved and thus listed in the Protein Data Bank. We expect that these database mapping issues may hinder the use of LUCApedia at the level of individual proteins, but they will mostly be mitigated once the predictions from individual proteins are aggregated into protein families, because missing annotations in one protein are likely to be present in other proteins within the same family.

### 2.1 Prebiotic chemistry datasets

The Prebiotic Chemistry datasets were categorized as such because they represent some aspect of a potential prebiotic chemistry that may be reflected in extant proteins. The four Prebiotic Chemistry datasets are composed of two hypothesis-based datasets and two empirical datasets from the same study (Goldford *et al.* 2017).

*Iron-sulfur clusters (12278 Uniprot accessions, 303 EggNOG accessions)*: Iron-sulfur cofactors have been previously proposed to reflect the potential role of iron-sulfur mineral surfaces as a possible setting for the origin of life and an important catalyst for very early life forms (Wächtershäuser 1988, Huber and Wächtershäuser 1997). This dataset is composed of all proteins that use at least one iron-sulfur cofactor.

*Zinc cofactors (22148 Uniprot accessions, 448 EggNOG accessions)*: Previous publications have proposed that zinc and zinc-sulfide catalysts may have played an important role in early nucleic acid chemistry and energy metabolism (Mulkidjanian 2009) and that the use of zinc cofactors in extant proteins reflects this early role in prebiotic chemistry (Mulkidjanian and Galperin 2009). This dataset is composed of all proteins that use at least one zinc cofactor.

*Goldford (8602 Uniprot accessions, 204 EggNOG accessions)*: A previous study by Goldford and colleagues generated a phosphate-free metabolic network by starting with simple prebiotically-available compounds and using a network expansion algorithm that assumed the evolution of catalysts or enzymes that could convert available metabolites into new metabolites (Goldford *et al.* 2017). The dataset is composed of all proteins with enzymatic functions matching those present in this hypothetical metabolic network.

*Goldford-thio (26255 Uniprot accessions, 558 EggNOG accessions)*: The minimal phosphate-free metabolic network developed by Goldford and colleagues assumed that only energetically favorable reactions could take place without the presence of ATP as an energy currency. The Goldford-thio dataset is composed of a larger network produced in the same study in which prebiotically-plausible thioesters (De Duve 1991) were used as an energy currency that predated ATP. The dataset is composed of all proteins with enzymatic functions matching those present in this expanded hypothetical metabolic network.

### 2.2 RNA world datasets

The RNA World category comprises datasets that reflect some aspect of either an RNA World stage in the evolution of the genetic system or an RNA-protein stage that would have followed it. The four RNA World datasets are composed of three hypothesis-based datasets and one study-based dataset.

*Ribozyme function analogs (6866 Uniprot accessions, 77 EggNOG accessions)*: The RNA World hypothesis has motivated synthetic biochemists to develop an increasingly broad array of ribozymes (i.e. catalytic RNAs) that can perform enzymatic functions now performed by proteins. This dataset is composed of proteins that perform a function that can also be performed by a natural or artificial ribozyme. The dataset was created by reviewing ribozyme literature, assigning Enzyme Commission (EC) numbers (Webb 1992, Bairoch 2000) to the ribozyme function, and identifying protein enzymes associated with the same EC numbers.

*Nucleotide cofactors (8647 Uniprot accessions, 242 EggNOG accessions)*: Many of the most important coenzymes in metabolism, e.g. ATP, NADH, Coenzyme A, and S-Adenosyl Methionine, are composed of or derived from nucleotides (White 1976). This observation led to the hypothesis that these coenzymes are remnants of earlier RNA World catalysts. This dataset is composed of all proteins that use these coenzymes (Kyrpides and Ouzounis 1995).

*Amino acid cofactors (1402 Uniprot accessions, 57 EggNOG accessions)*: The use of amino acids as ribozyme cofactors was previously proposed as a preadaptation that facilitated the origin of the genetic code. Specifically, sequences of nucleotides that bound amino acid cofactors could have evolved into the codons and anticodons that now comprise the genetic code (Szathmary 1999). This dataset is composed of all proteins that use cofactors derived from amino acids.

*RNA aptamers (17 Uniprot accessions, 6 EggNOG accessions)*: Blanco *et al.* (2018) identified proteins with known structures that bound artificial RNA aptamers and used these data to better characterize RNA-protein binding and the types of amino acids that are typically found in such interactions. This dataset is composed of the proteins used in this study.

### 2.3 LUCA protein domains

Nine different datasets are derived from empirical studies aimed at reconstructing features of the proteome of the LUCA. These study-based datasets have been split into two separate categories, LUCA Protein Domains and LUCA Protein Families. The LUCA Protein Domain datasets are derived from studies that focused on identifying universal protein domains, motifs, or structural folds. Most extant proteins have multiple domains, and related domains are often found in otherwise unrelated proteins (Chothia *et al.* 2003). However, it is thought that the earliest proteins were composed of single domains (Wang and Caetano-Anolles 2009). Four of the nine LUCA proteome studies represented in the LUCApedia database focus specifically on protein domains, motifs, or structural folds rather than complete proteins. The LUCA protein domain datasets all comprise proteins that contain at least one of the conserved domains identified in each respective study.

*Yang (2448 Uniprot accessions, 560 EggNOG accessions)*: Yang *et al.* (2005) developed a species phylogeny based on patterns of the presence or absence of protein fold superfamilies as catalogued in the SCOP database (Murzin *et al.* 1995). They identified 66 SCOP superfamilies that were present in all proteomes, which they consider to be the set of protein domains inherited from the proteome of the LUCA.

*Wang (8681 Uniprot accessions, 2024 EggNOG accessions)*: Wang *et al.* (2007) created a phylogeny of protein structure folds based on accessions in the SCOP database (Murzin *et al.* 1995). Phylogenetic relationships were determined based on both the taxonomic breadth of each fold and its frequency within individual proteomes. This phylogeny was used to identify 165 SCOP folds that emerged prior to the LUCA.

*Delaye (3646 Uniprot accessions, 440 EggNOG accessions)*: Delaye *et al.* (2005) identified protein domains and motifs, as defined by the Pfam database (Punta *et al.* 2012), that were found to be universal across bacterial and archaeal proteomes. The study identified 115 such universal Pfam domains and motifs.

*Ranea (42385 Uniprot accessions, 3861 EggNOG accessions)*: Ranea *et al.* (2006) used structural comparisons of proteins across bacteria and archaea to identify universal ancestral domain superfamilies as defined by the CATH database (Knudsen and Wiuf 2010, Sillitoe *et al.* 2021). The study identified 114 CATH domain superfamilies to have been present in the LUCA proteome.

### 2.4 LUCA protein families

Five additional study-based datasets are placed in the LUCA Protein Family category because they identified either protein families or protein functions that could be attributed to the proteome of the LUCA.

*Harris (29085 Uniprot accessions, 93 EggNOG accessions)*: Harris *et al.* (2003) surveyed clusters of proteins defined by the now defunct COG database (Tatusov *et al.* 1997) to identify protein families that were both universal and vertically inherited. Vertical inheritance was determined by comparison to an rRNA-based species tree. The study identified 80 such COGs that were attributed to the LUCA.

*Mirkin (97814 Uniprot accessions, 696 EggNOG accessions)*: Mirkin *et al.* (2003) performed an analysis similar to that of Harris et al., but included a model for gene loss, which led to a more permissive set of COGs being attributed to the LUCA. While several LUCA proteome models were reported in the study based on different gene gain/loss parameters, this dataset is based on the set of 572 COGs that was determined in the study to approximate a viable organism.

*Srinivasan (61163 Uniprot accessions, 932 EggNOG accessions)*: Srinivasan and Morowitz (2009) compared enzymatic reactions across several bacterial and archaeal metabolic networks cataloged in the KEGG database (Kanehisa and Goto 2000) to identify 286 shared reactions, deemed by this study to be universal metabolic reactions.

*Weiss (22292 UniProt accessions, 305 EggNOG accessions)*: Weiss *et al.* (2016) identified 355 protein families predicted to have been present in the proteome of the LUCA. Protein families were defined based on clusters of homologous proteins represented in the EggNOG database. LUCA ancestry was determined based on whether members of the protein family were found in at least two phyla from archaea and two phyla from bacteria, and also had tree topologies in which the bacterial clades and archaeal clades were respectively monophyletic.

*Moody (18723 Uniprot accessions, 264 EggNOG accessions)*: Moody *et al.* (2024) used gene-tree species-tree reconciliation based on the Amalgamated Likelihood Estimation algorithm (Szollosi *et al.* 2013) to generate probability scores that protein families were present in the proteome of the LUCA. Protein families were defined by the KEGG Orthology (KO) database (Kanehisa *et al.* 2016). The 399 protein families included in this LUCApedia dataset constitute the protein families with a LUCA probability score above 75% and taxonomic representation in both the bacterial and archaeal domains.

## 3 The LUCApedia web server

**Figure 2.**
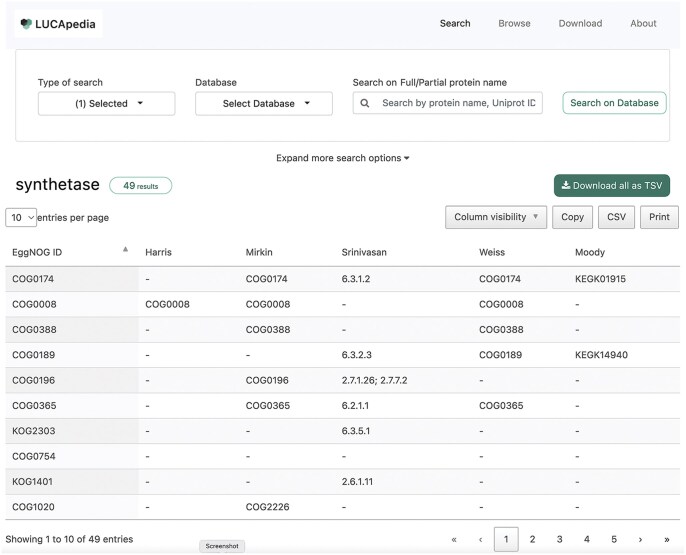
A screenshot of the LUCApedia web server. The image shows a results page following a search using the partial protein term “synthetase.” The search was performed on the EggNOG-based version of the database with only the five datasets from the “LUCA Protein Family Datasets” selected. Each row in the results table represents an EggNOG accession and each column represents a dataset based on one of the studies described above. Accessions in each cell in the results table, such as “6.3.2.3” (an EC number) or KEGK14940 (a KEGG Orthology ID), indicates the aspect of the EggNOG cluster that was predicted by the study or hypothesis to have been ancient. A dash represents no prediction.

The LUCApedia database can be downloaded and accessed as a web server through the URL, https://lucapedia.org/. The web server has both a search function and a browse function. The search function allows users to search for complete or partial protein names, UniProt IDs, EggNOG IDs, or Gene Ontology IDs (Ashburner *et al.* 2000, Gene Ontology *et al.* 2023). Either the UniProt-based version of the database or the EggNOG-based version of the database can be searched separately. While a default search shows results for all 17 datasets, these datasets can be individually selected either on the search page or the search results page. The results of a search (Fig. 2) are shown on a separate page and can be downloaded as a TSV file.

In addition to searching the LUCApedia database, users can also browse the database by the associated functions of the proteins or protein families. These functions are based on the Gene Ontology IDs linked to each LUCApedia entry. The Gene Ontology Database (Ashburner *et al.* 2000, Gene Ontology *et al.* 2023) characterizes proteins with respect to their molecular function, cellular location, and the biological process in which they participate. Gene Ontology terms describe these features of proteins at different levels of specificity and terms that are nested within other terms. The LUCApedia web server allows users to browse Gene Ontology terms from the most general level to the most specific level in a manner similar to the AmiGO web server (Carbon *et al.* 2009). When a user clicks on a GO term, they are presented with LUCApedia results associated with that GO term.

The web server also features a Download page where the UniProt-based version of the database and the EggNOG-based version of the database can be downloaded as TSV files. The documentation can also be accessed from this page or downloaded directly from the URL, https://lucapedia.org/assets/files/LUCApedia_documentation.pdf.

## 4 Conclusion

Since the beginning of the genomic era, computational biology has played a central role in the study of early evolution and especially the proteome of the LUCA (Kyrpides *et al.* 1999, Delaye 2024). Following its publication in 2013 (Goldman *et al.* 2013), the LUCApedia database has been used by a number of labs to study different aspects of early evolutionary history (Goldman *et al.* 2016, Goldford *et al.* 2017, Blanco *et al.* 2018, Crapitto *et al.* 2022). Here we present a new expanded LUCApedia database. Datasets that were present in the original database have been reconstituted with up-to-date database mapping; new datasets have been added to encompass a broader range of early evolutionary history; and LUCApedia entries have been aggregated into protein families to better facilitate comparison and corroboration between datasets.

This release of the new LUCApedia database coincides with an acceleration in the progress of computational biology techniques due to the implementation of increasingly sophisticated artificial intelligence strategies (Jumper *et al.* 2021, Dauparas *et al.* 2022). Anticipated developments in computational hardware may still further increase the efficacy of computational biology (Nalecz-Charkiewicz *et al.* 2024, Pyzer-Knapp and Curioni 2024). It is our hope that the LUCApedia database will provide a useful resource for the early evolution research community to take advantage of these advances and make new discoveries about the ancient history of life.

## Data Availability

All data discussed in this manuscript are available for download from the LUCApedia database web server https://lucapedia.org/data.

## References

[vbaf309-B1] Ashburner M , Ball CA, Blake JA et al Gene ontology: tool for the unification of biology. The gene ontology consortium. Nat Genet 2000;25:25–9. 10802651 10.1038/75556 PMC3037419

[vbaf309-B2] Bairoch A. The ENZYME database in 2000. Nucleic Acids Res 2000;28:304–5. 10592255 10.1093/nar/28.1.304 PMC102465

[vbaf309-B3] Baross JA , Hoffman SE. Submarine hydrothermal vents and associated gradient environments as sites for the origin and evolution of life. Origins Life Evol Biosphere 1985;15:327–45.

[vbaf309-B4] Becerra A , Delaye L, Islas S et al The very early stages of biological evolution and the nature of the last common ancestor of the three major cell domains. Annu Rev Ecol Evol Syst 2007;38:361–79.

[vbaf309-B5] Betts HC , Puttick MN, Clark JW et al Integrated genomic and fossil evidence illuminates life’s early evolution and eukaryote origin. Nat Ecol Evol 2018;2:1556–62. 30127539 10.1038/s41559-018-0644-x PMC6152910

[vbaf309-B6] Blanco C , Bayas M, Yan F et al Analysis of evolutionarily independent protein-RNA complexes yields a criterion to evaluate the relevance of prebiotic scenarios. Curr Biol 2018;28:526–37.e5. e525. 29398222 10.1016/j.cub.2018.01.014

[vbaf309-B7] Boeckmann B , Bairoch A, Apweiler R et al The SWISS-PROT protein knowledgebase and its supplement TrEMBL in 2003. Nucleic Acids Res 2003;31:365–70. 12520024 10.1093/nar/gkg095 PMC165542

[vbaf309-B8] Bowman JC , Hud NV, Williams LD. The ribosome challenge to the RNA world. J Mol Evol 2015;80:143–61. 25739364 10.1007/s00239-015-9669-9

[vbaf309-B9] Carbon S , Ireland A, Mungall CJ et al; Web Presence Working Group. AmiGO: online access to ontology and annotation data. Bioinformatics 2009;25:288–9. 19033274 10.1093/bioinformatics/btn615 PMC2639003

[vbaf309-B10] Cech TR. Ribozymes, the first 20 years. Biochem Soc Trans 2002;30:1162–6. 12440996 10.1042/bst0301162

[vbaf309-B11] Chothia C , Gough J, Vogel C et al Evolution of the protein repertoire. Science 2003;300:1701–3. 12805536 10.1126/science.1085371

[vbaf309-B12] Chu XY , Zhang HY. Cofactors as molecular fossils to trace the origin and evolution of proteins. Chembiochem 2020;21:3161–8. 32515532 10.1002/cbic.202000027

[vbaf309-B13] Crapitto AJ , Campbell A, Harris AJ et al A consensus view of the proteome of the last universal common ancestor. Ecol Evol 2022;12:e8930. 35784055 10.1002/ece3.8930 PMC9165204

[vbaf309-B14] Crick FH. The origin of the genetic code. J Mol Biol 1968;38:367–79. 4887876 10.1016/0022-2836(68)90392-6

[vbaf309-B15] Dauparas J , Anishchenko I, Bennett N et al Robust deep learning-based protein sequence design using ProteinMPNN. Science 2022;378:49–56. 36108050 10.1126/science.add2187 PMC9997061

[vbaf309-B16] De Duve C. Did god make RNA? Nature 1988;336:209–10. 2461514 10.1038/336209b0

[vbaf309-B17] De Duve C. Blueprint for a Cell: The Nature and Origin of Life. Burlington, NC: N. Patterson, 1991.

[vbaf309-B18] Delaye L. The unfinished reconstructed nature of the last universal common ancestor. J Mol Evol. 2024;92:584–92. 39026043 10.1007/s00239-024-10187-8 PMC11458799

[vbaf309-B19] Delaye L , Becerra A, Lazcano A. The last common ancestor: what’s in a name? Orig Life Evol Biosph 2005;35:537–54. 16254691 10.1007/s11084-005-5760-3

[vbaf309-B20] Fer E , Yao T, McGrath KM et al The origins and evolution of translation factors. Trends Genet 2025;41:590–600. 40133153 10.1016/j.tig.2025.02.004 PMC12237606

[vbaf309-B21] Ferris JP , Edelson EH, Mount NM et al The effect of clays on the oligomerization of HCN. J Mol Evol 1979;13:317–30. 229235 10.1007/BF01731372

[vbaf309-B22] Ferris JP , Hill AR, Liu R et al Synthesis of long prebiotic oligomers on mineral surfaces. Nature 1996;381:59–61. 8609988 10.1038/381059a0

[vbaf309-B23] Fournier GP , Andam CP, Alm EJ et al Molecular evolution of aminoacyl tRNA synthetase proteins in the early history of life. Orig Life Evol Biosph 2011;41:621–32. 22200905 10.1007/s11084-011-9261-2

[vbaf309-B24] Freeland SJ , Knight RD, Landweber LF. Do proteins predate DNA? Science 1999;286:690–2. 10577226 10.1126/science.286.5440.690

[vbaf309-B25] Gene Ontology Consortium. The gene ontology knowledgebase in 2023. Genetics 2023;224:iyad031. 10.1093/genetics/iyad031. 36866529 PMC10158837

[vbaf309-B26] Gilbert W. Origin of life: the RNA world. Nature 1986;319:618.

[vbaf309-B27] Goldford JE , Hartman H, Smith TF et al Remnants of an ancient metabolism without phosphate. Cell 2017;168:1126–34.e9. 28262353 10.1016/j.cell.2017.02.001

[vbaf309-B28] Goldman AD , Baross JA, Samudrala R. The enzymatic and metabolic capabilities of early life. PLoS One 2012;7:e39912. 22970111 10.1371/journal.pone.0039912 PMC3438178

[vbaf309-B29] Goldman AD , Beatty JT, Landweber LF. The TIM barrel architecture facilitated the early evolution of protein-mediated metabolism. J Mol Evol 2016;82:17–26. 26733481 10.1007/s00239-015-9722-8 PMC4709378

[vbaf309-B30] Goldman AD , Bernhard TM, Dolzhenko E et al LUCApedia: a database for the study of ancient life. Nucleic Acids Res 2013;41:D1079–82. 23193296 10.1093/nar/gks1217 PMC3531223

[vbaf309-B31] Goldman AD , Fournier GP. The very early evolution of biological complexity. Trends Genet 2024;40:912–3. 39327101 10.1016/j.tig.2024.09.001

[vbaf309-B32] Goldman AD , Kacar B. Cofactors are remnants of life’s origin and early evolution. J Mol Evol 2021;89:127–33. 33547911 10.1007/s00239-020-09988-4 PMC7982383

[vbaf309-B33] Goldman AD , Landweber LF. Oxytricha as a modern analog of ancient genome evolution. Trends Genet 2012;28:382–8. 22622227 10.1016/j.tig.2012.03.010 PMC3401270

[vbaf309-B34] Goldman AD , Samudrala R, Baross JA. The evolution and functional repertoire of translation proteins following the origin of life. Biol Direct 2010;5:15. 20377891 10.1186/1745-6150-5-15 PMC2873265

[vbaf309-B35] Gribaldo S , Cammarano P. The root of the universal tree of life inferred from anciently duplicated genes encoding components of the protein-targeting machinery. J Mol Evol 1998;47:508–16. 9797401 10.1007/pl00006407

[vbaf309-B36] Harris AJ , Goldman AD. The very early evolution of protein translocation across membranes. PLoS Comput Biol 2021;17:e1008623. 33684113 10.1371/journal.pcbi.1008623 PMC7987157

[vbaf309-B37] Harris JK , Kelley ST, Spiegelman GB et al The genetic core of the universal ancestor. Genome Res 2003;13:407–12. 12618371 10.1101/gr.652803 PMC430263

[vbaf309-B38] Hsiao C , Mohan S, Kalahar BK et al Peeling the onion: ribosomes are ancient molecular fossils. Mol Biol Evol 2009;26:2415–25. 19628620 10.1093/molbev/msp163

[vbaf309-B39] Huang H , McGarvey PB, Suzek BE et al A comprehensive protein-centric ID mapping service for molecular data integration. Bioinformatics 2011;27:1190–1. 21478197 10.1093/bioinformatics/btr101 PMC3072559

[vbaf309-B40] Huber C , Wächtershäuser G. Activated acetic acid by carbon fixation on (Fe, Ni)S under primordial conditions. Science 1997;276:245–7. 9092471 10.1126/science.276.5310.245

[vbaf309-B41] Huerta-Cepas J , Szklarczyk D, Heller D et al eggNOG 5.0: a hierarchical, functionally and phylogenetically annotated orthology resource based on 5090 organisms and 2502 viruses. Nucleic Acids Res 2019;47:D309–14. 30418610 10.1093/nar/gky1085 PMC6324079

[vbaf309-B42] Jerome CA , Kim H-J, Mojzsis SJ et al Catalytic synthesis of polyribonucleic acid on prebiotic rock glasses. Astrobiology 2022;22:629–36. 35588195 10.1089/ast.2022.0027 PMC9233534

[vbaf309-B43] Jumper J , Evans R, Pritzel A et al Highly accurate protein structure prediction with AlphaFold. Nature 2021;596:583–9. 34265844 10.1038/s41586-021-03819-2 PMC8371605

[vbaf309-B44] Kailing F , Lieberman J, Wang J et al Evolution of cellular organization along the first branches of the tree of life. J Mol Evol 2024;92:618–23. 39020132 10.1007/s00239-024-10188-7 PMC11458647

[vbaf309-B45] Kanehisa M , Goto S. KEGG: kyoto encyclopedia of genes and genomes. Nucleic Acids Res 2000;28:27–30. 10592173 10.1093/nar/28.1.27 PMC102409

[vbaf309-B46] Kanehisa M , Sato Y, Kawashima M et al KEGG as a reference resource for gene and protein annotation. Nucleic Acids Res. 2016;44:D457–62. 26476454 10.1093/nar/gkv1070 PMC4702792

[vbaf309-B47] Kirschning A. Coenzymes and their role in the evolution of life. Angew Chem Int Ed Engl 2021;60:6242–69. 31945250 10.1002/anie.201914786 PMC7983987

[vbaf309-B48] Knudsen M , Wiuf C. The CATH database. Hum Genomics 2010;4:207–12. 20368142 10.1186/1479-7364-4-3-207 PMC3525972

[vbaf309-B49] Kyrpides N , Overbeek R, Ouzounis C. Universal protein families and the functional content of the last universal common ancestor. J Mol Evol 1999;49:413–23. 10485999 10.1007/pl00006564

[vbaf309-B50] Kyrpides NC , Ouzounis CA. Nucleic acid-binding metabolic enzymes: living fossils of stereochemical interactions? J Mol Evol 1995;40:564–9. 7543949 10.1007/BF00160502

[vbaf309-B51] Lai YC , Liu Z, Chen IA. Encapsulation of ribozymes inside model protocells leads to faster evolutionary adaptation. Proc Natl Acad Sci USA 2021;118:e2025054118. 34001592 10.1073/pnas.2025054118 PMC8166191

[vbaf309-B52] Ledford SM , Meredith LK. Volatile organic compound metabolism on early earth. J Mol Evol 2024;92:605–17. 39017923 10.1007/s00239-024-10184-x PMC11458752

[vbaf309-B53] Levy M , Miller SL, Brinton K et al Prebiotic synthesis of adenine and amino acids under europa-like conditions. Icarus 2000;145:609–13. 11543508 10.1006/icar.2000.6365

[vbaf309-B54] Martin W , Baross J, Kelley D et al Hydrothermal vents and the origin of life. Nat Rev Microbiol 2008;6:805–14. 18820700 10.1038/nrmicro1991

[vbaf309-B55] Menor-Salvan C et al Synthesis of pyrimidines and triazines in ice: implications for the prebiotic chemistry of nucleobases. Chem Eur J 2009;15:4411–8. 19288488 10.1002/chem.200802656

[vbaf309-B56] Mirkin BG , Fenner TI, Galperin MY et al Algorithms for computing parsimonious evolutionary scenarios for genome evolution, the last universal common ancestor and dominance of horizontal gene transfer in the evolution of prokaryotes. BMC Evol Biol 2003;3:2. 12515582 10.1186/1471-2148-3-2 PMC149225

[vbaf309-B57] Moody ERR , Álvarez-Carretero S, Mahendrarajah TA et al The nature of the last universal common ancestor and its impact on the early earth system. Nat Ecol Evol 2024;8:1654–66. 38997462 10.1038/s41559-024-02461-1 PMC11383801

[vbaf309-B58] Mulkidjanian AY. On the origin of life in the zinc world: 1. Photosynthesizing, porous edifices built of hydrothermally precipitated zinc sulfide as cradles of life on earth. Biol Direct 2009;4:26. 10.1186/1745-6150-4-26. 19703272 PMC3152778

[vbaf309-B59] Mulkidjanian AY , Galperin MY. On the origin of life in the zinc world. 2. Validation of the hypothesis on the photosynthesizing zinc sulfide edifices as cradles of life on earth. Biol Direct 2009;4:27. 10.1186/1745-6150-4-27. 19703275 PMC2749021

[vbaf309-B60] Murzin AG , Brenner SE, Hubbard T et al SCOP: a structural classification of proteins database for the investigation of sequences and structures. J Mol Biol 1995;247:536–40. 7723011 10.1006/jmbi.1995.0159

[vbaf309-B61] Nalecz-Charkiewicz K , Charkiewicz K, Nowak RM. Quantum computing in bioinformatics: a systematic review mapping. Brief Bioinform 2024;25:bbae391. 39140856 10.1093/bib/bbae391 PMC11323091

[vbaf309-B62] Punta M , Coggill PC, Eberhardt RY et al The pfam protein families database. Nucleic Acids Res. 2012;40:D290–301. 22127870 10.1093/nar/gkr1065 PMC3245129

[vbaf309-B63] Pyzer-Knapp EO , Curioni A. Advancing biomolecular simulation through exascale HPC, AI and quantum computing. Curr Opin Struct Biol 2024;87:102826. 38733863 10.1016/j.sbi.2024.102826

[vbaf309-B64] Ranea JAG , Sillero A, Thornton JM et al Protein superfamily evolution and the last universal common ancestor (LUCA). J Mol Evol 2006;63:513–25. 17021929 10.1007/s00239-005-0289-7

[vbaf309-B65] Rivas M , Becerra A, Peretó J et al Metalloproteins and the pyrite-based origin of life: a critical assessment. Orig Life Evol Biosph 2011;41:347–56. 21431891 10.1007/s11084-011-9238-1

[vbaf309-B66] Rivas M , Fox GE. How to build a protoribosome: structural insights from the first protoribosome constructs that have proven to be catalytically active. RNA 2023;29:263–72. 36604112 10.1261/rna.079417.122 PMC9945445

[vbaf309-B67] Rivas M , Fox GE. On the nature of the last common ancestor: a story from its translation machinery. J Mol Evol 2024;92:593–604. 39259330 10.1007/s00239-024-10199-4

[vbaf309-B68] Russell MJ , Hall AJ. The emergence of life from iron monosulphide bubbles at a submarine hydrothermal redox and pH front. J Geol Soc London 1997;154:377–402. 10.1144/gsjgs.154.3.0377 11541234

[vbaf309-B69] Sillitoe I , Bordin N, Dawson N et al CATH: increased structural coverage of functional space. Nucleic Acids Res 2021;49:D266–73. 33237325 10.1093/nar/gkaa1079 PMC7778904

[vbaf309-B70] Srinivasan V , Morowitz HJ. The canonical network of autotrophic intermediary metabolism: minimal metabolome of a reductive chemoautotroph. Biol Bull 2009;216:126–30. 19366923 10.1086/BBLv216n2p126

[vbaf309-B71] Szathmary E. The origin of the genetic code: amino acids as cofactors in an RNA world. Trends Genet 1999;15:223–9. 10354582 10.1016/s0168-9525(99)01730-8

[vbaf309-B72] Szollosi GJ et al Efficient exploration of the space of reconciled gene trees. Syst Biol 2013;62:901–12. 23925510 10.1093/sysbio/syt054 PMC3797637

[vbaf309-B73] Tatusov RL , Koonin EV, Lipman DJ. A genomic perspective on protein families. Science 1997;278:631–7. 9381173 10.1126/science.278.5338.631

[vbaf309-B74] UniProt Consortium. UniProt: the universal protein knowledgebase in 2023. Nucleic Acids Res 2023;51:D523–31. 36408920 10.1093/nar/gkac1052 PMC9825514

[vbaf309-B75] Visser CM. Evolution of biocatalysis 1. Possible pre-genetic-code RNA catalysts which are their own replicase. Orig Life 1984;14:291–300. 6205343 10.1007/BF00933670

[vbaf309-B76] Wächtershäuser G. Before enzymes and templates: theory of surface metabolism. Microbiol Rev 1988;52:452–84. 3070320 10.1128/mr.52.4.452-484.1988 PMC373159

[vbaf309-B77] Wang M , Caetano-Anolles G. The evolutionary mechanics of domain organization in proteomes and the rise of modularity in the protein world. Structure 2009;17:66–78. 19141283 10.1016/j.str.2008.11.008

[vbaf309-B78] Wang M , Yafremava LS, Caetano-Anollés D et al Reductive evolution of architectural repertoires in proteomes and the birth of the tripartite world. Genome Res 2007;17:1572–85. 17908824 10.1101/gr.6454307 PMC2045140

[vbaf309-B79] Webb EC. Enzyme Nomenclature 1992: Recommendations of the Nomenclature Committee of the International Union of Biochemistry and Molecular Biology and the Nomenclature and Classification of Enzymes. London: Elsevier Science, 1992.

[vbaf309-B80] Weiss MC , Sousa FL, Mrnjavac N et al The physiology and habitat of the last universal common ancestor. Nat Microbiol 2016;1:16116. 27562259 10.1038/nmicrobiol.2016.116

[vbaf309-B81] White HB III. Coenzymes as fossils of an earlier metabolic state. J Mol Evol 1976;7:101–4. 1263263 10.1007/BF01732468

[vbaf309-B82] Woese C. The universal ancestor. Proc Natl Acad Sci USA 1998;95:6854–9. 9618502 10.1073/pnas.95.12.6854 PMC22660

[vbaf309-B83] Yang S , Doolittle RF, Bourne PE. Phylogeny determined by protein domain content. Proc Natl Acad Sci USA 2005;102:373–8. 15630082 10.1073/pnas.0408810102 PMC540256

